# Iatrogenic endometriosis following apical pelvic organ prolapse surgery: a case report

**DOI:** 10.1186/s13256-019-2327-x

**Published:** 2020-01-05

**Authors:** Alkan Cubuk, Orkunt Ozkaptan, Jörg Neymeyer

**Affiliations:** 1 Department of Urology, Kartal Dr. Lütfi Kırdar Traning and Research Hospital, Istanbul, Turkey; 2 Department of Urology, Charité Universitätsmedizin Berlin, Berlin, Germany

**Keywords:** Endometriosis, Pelvic organ prolapse, Laparoscopy, Hysterectomy

## Abstract

**Background:**

Iatrogenic endometriosis is the presence of endometrial glands and stroma out of the uterus following certain surgical interventions. The rate of iatrogenic endometriosis after gynecologic surgeries due to benign uterine disease is 1–2%. Laparoscopic supracervical hysterectomy is also a part of frequently used surgical treatment of apical pelvic organ prolapse, which is followed by sacrocervicopexy. However, there are no data about iatrogenic endometriosis after apical prolapse surgery in the current literature. Herein, we present a case report of a patient diagnosed with *de novo* endometriosis 1 year after laparoscopic supracervical hysterectomy and sacrocervicopexy.

**Case presentation:**

A 46-year-old parous Slavic woman who underwent laparoscopic supracervical hysterectomy and sacrocervicopexy secondary to grade 3 symptomatic apical prolapse 1 year earlier was admitted to the same clinic with pelvic pain that had started 6 months following surgery. Deep vaginal palpation was painful. Transvaginal ultrasonography revealed an area with hypervascularization on the sacral promontory. She was scheduled for diagnostic laparoscopy. A 2 × 2-cm solid, wine-colored, hypervascular hemorrhagic lesion was seen on the sacral promontory. The lesion and the peritoneal layer behind it were totally excised. The patient was discharged on the first postoperative day, without any complications. Pathologic examination revealed foci of endometriosis comprising endometrial glands and stroma within the connective tissue, along with hemosiderin-laden macrophages. The symptoms of the patient resolved after the surgery, and no further adjuvant treatment was needed.

**Conclusion:**

Although the rate of iatrogenic endometriosis is low after laparoscopic supracervical hysterectomy and sacrocervicopexy, the possibility of the occurrence of iatrogenic endometriosis should be discussed with patients who are diagnosed with apical prolapse to determine the type of surgical intervention. Iatrogenic endometriosis should be kept in mind for differential diagnosis in case of pain after laparoscopic supracervical hysterectomy and sacrocervicopexy.

## Introduction

Iatrogenic endometriosis (IE) is the presence of endometrial glands and stroma out of the uterus following certain surgical interventions, such as total or supracervical hysterectomy, myomectomy, and cesarean section [1]. The most common localizations for IE are cesarean scares (skin scare, uterus scare), trocar sites, sigmoid colon, ovaries, bladder, vaginal vault, and the parietal peritoneum [2, 3]. Pain, dyspareunia, bleeding, and palpable mass are the common symptoms of IE. The interval between previous surgery and occurrence of symptoms ranges between first menstruation time and 7 years after surgery [4]. IE is reported with a rate of 1.4% after hysterectomy [5]. Data on IE is spare and comes from case reports and small case series. All these publications report series of gynecologic surgery cases due to benign diseases such as adenomyosis, uterine leiomyomas, and fibroids [1, 5]. Supracervical hysterectomy is also a frequently used surgical treatment for apical pelvic organ prolapse (POP), which is followed by sacrocervicopexy [6]. However, there are no data on IE after apical prolapse surgery in the current literature. Herein, we present a case report of a patient diagnosed with *de novo* endometriosis 1 year after laparoscopic supracervical hysterectomy and sacrocervicopexy (LASH). To the best of our knowledge, this is the first such case report in the literature.

## Case presentation

### Patient

A 46-year-old parous Slavic woman who underwent LASH secondary to grade 3 symptomatic apical prolapse 1 year ago was admitted to our clinic with pelvic pain that had started 6 months following surgery. It worsened with sexual intercourse and was not relieved with oral analgesic drugs. She had no complaints of urinary incontinence or prolapse. She did not have any remarkable bladder or bowel symptoms. On physical examination with vaginal speculum, pain upon deep vaginal palpation was detected, but there was no sign of erosion on the cervix and vaginal wall. Transvaginal ultrasonography (US) revealed a hypoechogenic and hypervascular solid area with irregular contours sized 3 × 2 cm in diameter on the sacral promontory. Transabdominal US showed normal kidneys and ureters with normal peristalsis. A flexible cystoscopy and office-based rectosigmoidoscopy was performed to exclude any reason for pelvic pain. No further abnormal findings were observed in the bladder and rectosigmoid area. The patient was reluctant to undergo further diagnostic imaging such as pelvic magnetic resonance imaging (MRI) because of her insurance status. After discussing the diagnostic possibilities with the patient, she was scheduled for diagnostic laparoscopy.

### Surgical technique

An informed consent was obtained from the patient preoperatively. After administration of general anesthesia and antibiotic prophylaxis, the patient was placed in Trendelenburg position. A Veress needle was used to achieve pneumoperitoneum. Previous trocar sites were used for laparoscopic port placement; a 12-mm port was placed at the inferior margin of the umbilicus, and 5-mm ports were placed medially to iliac spine on both sides. After the port placement, adhesions secondary to the previous surgery were excised. The position of the mesh under peritoneum from promontory to cervical uteri was totally normal. No abnormalities were detected regarding ureters.

**Fig. 1:**
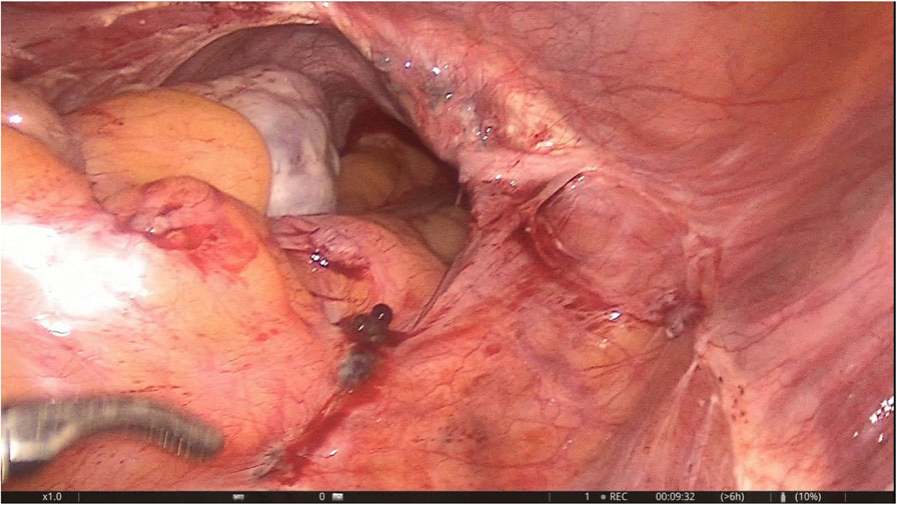
Endometriosis on sacral promontory

**Fig. 2:**
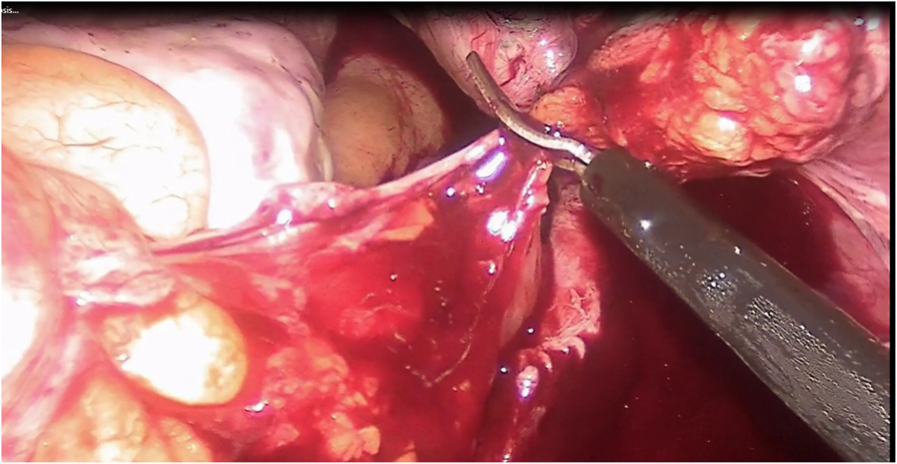
Excision of endometriosis with peritoneum

**Fig. 3:**
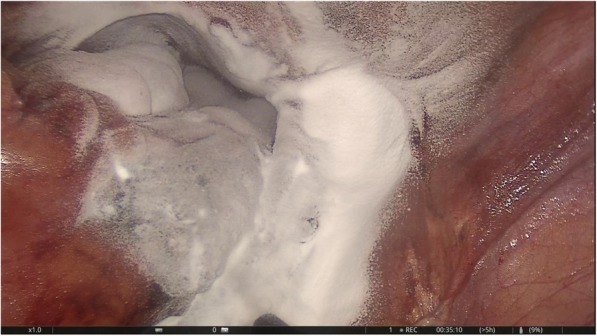
Final view

The only pathologic finding was detected on the peritoneum covering the sacral promontory: a 2 × 2-cm, solid, wine-colored, hypervascular hemorrhagic lesion, which macroscopically resembled endometriosis (Fig. 1). The lesion and the peritoneal layer behind it were totally excised (Fig. 2). There was no sign of invasion to the deeper parts of the pelvic wall and organs. The abdominal cavity was checked for other potential focal lesions of endometriosis and washed out with saline. After hemostasis was achieved using bipolar cautery and applying hemostatic agents, the procedure was terminated (Fig. 3). The patient was discharged on the first postoperative day, without any complications.

Pathologic examination revealed foci of endometriosis comprising endometrial glands and stroma within the connective tissue, along with hemosiderin-laden macrophages. The patient was classified as having stage 1 disease (minimal disease with few implants on the peritoneum) according to the American Society for Reproductive Medicine and category 1 (peritoneal endometriosis) according to the Endometriosis Foundation of America. The symptoms of the patient resolved, and no adjuvant treatment was needed up to 1 year after surgery. A clinical visit including physical examination and US assessment was planned for every 6 months as follow-up.

## Discussion

Endometriosis has been known since its first description by Sampson in 1924 as the presence of functional endometrial tissues out of the uterine cavity [7]. However, IE has been a subject of publications since the 1990s, after the popularization of laparoscopic and robotic total or supracervical hysterectomy procedures [2, 8].

The rate of IE was reported to be 1.4% in a case-control study by Schuster *et al.* [5]. Among 464 hysterectomy cases, the data of 16 patients who were reoperated due to pain and bleeding were evaluated, and five cases with IE were identified. In a review published by Pereira *et al*., 32 different publications were evaluated, and among 66 patients who underwent reoperation after hysterectomy, 4 with IE were identified [1]. In these two publications, indications of hysterectomies were benign uterine diseases, whereas no data are currently available about IE after prolapse surgery. According to our clinical records, we have only 1 case of IE but 15 reoperations after 600 LASH procedures. It seems that our IE rate is lower than in the studies mentioned before. The underlying reason for this uncertain rate of IE may be the limited number of cases in which reoperation is needed following total/subtotal hysterectomy, myomectomy, and LASH.

Hilger *et al.* suggested that in the presence of endometriosis and adenomyosis at the time of previous surgery, retrograde flow from resting endometrial tissues in the cervix is a reason for IE after supracervical hysterectomy [9]. Stefanović *et al*. emphasized direct endometrial tissue implantation and seeding on pathogenesis of IE, especially after cesarean section surgery and the morcellation of uteri [10]. We viewed our patient’s LASH surgery video record, and we did not see endometrial foci on the sacral promontory. Therefore, a missed endometrial lesion was not a possibility in our patient. Retrograde flow might also not be a reason for IE in our patient, owing to the closure of the peritoneum on the cervical stump. However, an early breakdown of sutures placed on the peritoneum above the cervix or laceration of the peritoneum might cause leakage from the cervix to the peritoneum, resulting in IE. The probable reasons for IE in our patient might be the seeding of endometrial cells secondary to morcellation and undiagnosed adenomyosis at the time of previous surgery.

Steiner *et al.* first described electric morcellation of the uterus for laparoscopic hysterectomy in 1993 [11]. Although it allows removal of the specimen without an incision, a lot of morcellator-related complications have been published in the literature. Milad *et al.* reported morcellator-related injuries in 55 cases and deaths in 6 cases [12]. Tulandi *et al*. described pathogenesis of parasitic leiomyomas and disseminated peritoneal leiomyomas secondary to morcellation [2]. Pereira *et al*. described cancer tissue spreading to the peritoneal cavity in a case with occult malignancy in the uterus after use of a morcellator [1]. In 2014, the U.S. Food and Drug Administration released a notification discouraging the use of electric morcellators [13]. After this notification, Solima *et al.* suggested using a confined morcellator with a specimen bag. They stated that leakage from the bag and increase in operation time were problems [14].

In our practice, we routinely check patients in terms of uterine malignancies and use nonconfined morcellation preoperatively. We did not experience any injury or death related to morcellator use, and also no occult uterine malignancies were detected. However, after our present patient’s case, we can argue that unless the specimen bag is damaged, confined morcellation with a bag can be a beneficial option to avoid IE. Also removing the specimen via an incision to avoid IE can be discussed with the patient preoperatively.

Kill *et al.* suggested that the most common symptoms of endometriosis are pain, dyspareunia, local mass effects, and bleeding [4]. In our patient’s case, pelvic pain was the main symptom, and pain during deep vaginal palpation was the main finding on physical examination. Bazot *et al*. stated that USG and MRI are the first-line diagnostic tools for endometriosis [15]. However, they remarked on the limitations of MRI as absence of international consensus on reporting IE and the sensitivity of MRI for lesions > 7 mm. The limitation of US is that the success rate of detecting IE is operator-dependent [15]. We determined hypervascularization and scare/fibrosis as findings of endometriosis on the basis of US. However, the localization of IE in our patient was the sacral promontory, and we thought these findings were secondary to mesh used during the previous LASH surgery.

In a review by Kızılay *et al*., it was suggested that the treatment of endometriosis aims at pain relief, preserving fertility, and preventing obstruction [7]. Oral contraceptive drugs, analgesic drugs, gonadotropin-releasing hormone agonists, and aromatase inhibitors are the first-line medical treatment options [3]. Surgical excision is usually necessary to preserve fertility and to treat urinary obstruction related to endometriosis [7]. When it comes to IE, there is no specific treatment algorithm in the literature; however, surgical excision has been discussed as the first treatment option by authors [3–5]. In accordance with the literature, diagnostic laparoscopy and excision of endometriosis was our preferred treatment modality.

## Conclusion

IE is a rare complication of total or supracervical hysterectomies for benign uterine diseases. The pathogenesis can be explained by the presence of endometriosis or adenomyosis at the time of previous surgery, retrograde flow from the rest of endometrial tissues, and directly implantation and seeding of endometrial tissue. IE can also be diagnosed secondary to laparoscopic supracervical hysterectomy and electrical morcellation of the uterus as a part of POP surgery. With the wide application of these procedures, an increase in the rates of IE following POP surgeries may be detected in the future. Although the rate of IE is low after LASH surgery, the possibility for the occurrence of IE should be discussed with patients who are diagnosed with apical prolapse to determine the type of surgical intervention. IE should be kept in mind for differential diagnosis in case of pain after LASH surgery. Laparoscopic intervention may provide the diagnosis and further the excision of IE. Further studies are needed to determine the real rates and proper treatment modalities of IE and eligible surgical maneuvers to avoid IE during prolapse surgery.

## Data Availability

The authors agree to make the images and data described in the manuscript freely available for use.
